# Silver Depreciation in 3-Polker Coins Issued during 1619–1627 by Sigismund III Vasa King of Poland

**DOI:** 10.3390/ma15217514

**Published:** 2022-10-26

**Authors:** Ioan Petean, Gertrud Alexandra Paltinean, Emanoil Pripon, Gheorghe Borodi, Lucian Barbu Tudoran

**Affiliations:** 1 Faculty of Chemistry and Chemical Engineering, Babes-Bolyai University, 11 Arany Janos Street, 400028 Cluj-Napoca, Romania; 2 Zalau County Museum of History and Art, 9 Unirii Street, 450042 Zalau, Romania; 3 National Institute for Research and Development of Isotopic and Molecular Technologies,65-103 Donath Street, 400293 Cluj-Napoca, Romania; 4 Faculty of Biology and Geology, Babes-Bolyai University, 44 Gheorghe Bilaşcu Street, 400015 Cluj-Napoca, Romania

**Keywords:** medieval coins, silver concentration, materials archeometry

## Abstract

The present research is focused on the 3-Polker coins issued during 1619–1627 by Sigismund III Vasa, King of Poland. A major financial crisis took place at that time due to the 30-year War, which started in 1619. There are two theories among historians concerning the silver depreciation of these coins. The most common theory (generally accepted without proof) is that the later years of issue are depreciated below 60% Ag. The second theory is based on the medieval sources that indicate inflation during the years from 1621–1625, but the medieval source only refers to the inflation of the type of coins and does not mention the issuer. Therefore, in this study, we use modern investigation techniques and materials science methods to help historians elucidate the aforementioned aspects regarding the medieval period. The XRD investigation results are in good agreement with the SEM-EDX elemental analysis. The coins from 1619 and 1620 have high silver content, namely, 86.97% and 92.49%, which corresponds to good silver. The amount of Ag found in the coins from 1621–1625 issituated in the range of 63.2–74.6%. The silver titleis suddenly restored in 1626 at about 84.3% and is kept in a good range until the end of this decree under Sigismund III in 1627. In conclusion, the second theory was partly validated by our experimental results, certifying the currency depreciation during 1621–1625, but the silver title was not lower than 54.2%. Notably, even this depreciated silver title assures a good quality of the 3-Polker coins compared to similar coins issued in other countries that were copper–silver-plated. Therefore, the 3-Polker coins were preferably hoarded at that time.Small alterations in the mint mark’s design were observed in all the depreciated coins compared to the good ones. This might be a sign for an expert to identify the depreciated coins, a fact which requires supplementary investigations. The silver title’s restoration in 1626 also came with a complete change of the mintmark.

## 1. Introduction

Many modern problems are similar to ones from the medieval and pre-modern ages, especially the financial crises that occur from time to time [1,2]. The medieval minters often hid the financial crises and currency inflation on the inscription MONETA NOVA (a Latin expression meaning new currency) etched on the obverse side along with the minting year [3]. This inscription, combined with the issue year, allows a medieval wise man to identify which coin is good and which is depreciated. Such knowledge assures the success of the wise traders in their business and in their dealings with tax collectors. Inflation occurs by the depreciation of the silver amount in the issued coins. Since all alloys containing at least 40% Ag are white and bright, it would be difficult for medieval people to know whether a certain coin has good title (at least 80% silver) or is depreciated.

The historical background of the 3-Polker coins issued by Sigismund III Vasa, King of Poland and Grand duke of Lithuania, is absolutely necessary for the accuracy of the present study. This is a small silver coin with a 19.8 mm diameter and a thickness of about 0.55 mm, weighting from 1.21 to 1.54 g depending on the minting year. It is also known under the common Polish name as Półtorak, which is equivalent to the Poltura (1.5 Kreutzer) from the Austrian Roman Empire [4]. Its denomination is 1/24 that of the German Thaler and is intended for large circulation. There are some inconstancies and arguments about their silver title. The Thaler was the silver standard for commercial trade, and it was not available in large circulation; rather, it was used in important commercial transactions. Therefore, the 3-Polker was spread widely among the peoples from the Polish–Lithuanian Commonwealth to the vicinity countries [4,5]. The scarce information from the original sources has raised two possible theories about the 3-Polker coins’ silver title and particularly about those who generated the inflation during 1620–1625.

The simplest theory is very popular among coin collectors and assumes the general opinion about coins’ evolution during the Pre-Modern Age where in the earliest coins are of a good silver quality and the latest are depreciated due to the inflation generated during their circulating period. Thus, the earliest 3-Polker coins issued by Sigismund III in 1614 should be made of good silver, which is expected to constantly decrease to the last year of theirproduction in 1627. Certain clues make this theory generally accepted by the collectors without scientific proofand only based on their own observations.

The more sophisticated theory is represented by the historians’ research into the sources ranging as close as possible to the minting time [5,6]. The oldest records regarding the 3-Polker coins issued by Sigismund III mention that in 1614, the initial silver title was about 46.9% purity with a net weight of 1.54 g, corresponding to 0.72 g of pure silver. The records mention that in 1619, the silver title decreased to only 40.6%purity, with a net weight of 1.21 g and a silver content of 0.49 g.

Starting from the second decade of the seventeenth century, the 3-Polker coins appear in the circulation of Transylvanian currency. These coins’ flux along with the depreciated issues of the Transylvanian Prince Gabriel Bethlen were considered as the causative agents of the inflation during 1620–1625 [4,6]. The Transylvanian Diet (e.g., legislative organization) held in Cluj in May 1622 settled an exchange course of 4 silver denars for the old Poltura, regardless of the issuing year, and of 2 denars for the issues minted in 1620 and after considering the abovementioned conditions. In the Transylvanian Diet held in Bistrita in the autumn of the same year, the exchange rate was modified to 4 denars for all good Poltura, regardless of the minting year, and the small Poltura were to be exchanged at a rate of 3 denars until the end of 1623 and after at a rate of 2 denars [6].

Nagy Szabó Ferencz of Târgu Mureș (1580–1658) mentions some medieval sources that discuss “copper-rich półtorak” having a silver title of about 12.5%, which came into Transylvania from Silesia [7]. Based on these scripts, some numismatists considered that the “copper rich Polturak” would have been the 3-Polker coins issued by Sigismund III of Poland, while others consider thisto be impossible [4]. The Hungarian historian BuzaJános remarks that the Polturak’s name was also used before 1614 for coins with a similar denomination as the 3-Polker such as the imperial coins of the 3 Kreutzer (1/24 of the Thaler’s gross value) [8]. Besides all the presented historical aspects regarding the 3-Polker coins’ quality, they were found in significant hoards discovered in Salaj and Cluj counties, which are now in the custody of the Zalau Museum and were reported in the following literature: Salajeni Hoard [9]; Verveghiu Hoard [10]; Zalau Hoard No. 1 [11]; Mineu Hoard [12]; Aghires Hoard [13].

It is relatively difficult to determine which is the true supposition between the collector’s beliefs and old historical records. Materials science-based research is required to fulfil this purpose. The recent literature reports modern material investigation techniques such as X-ray diffraction (XRD), X-ray photoelectron spectroscopy (XPS), and SEM-EDX, which have beenemployed to reveal the hidden secrets of metallic parts of medieval artefacts such as metallic parts within medieval clothes [14] and metallic fibres within burial robes from medieval crypts [15], and to investigate the surface alteration of metallic artefacts exposed to a closed environment [16].

An optimal match must be found between the artefact and the investigation methods to reveal the artefact’s secrets. In our case, regarding the 3-Polker coins, this concerns the Ag–Cu binary system, which is a system with total insolubility and that has an eutectic at the composition of 719 ‰ Ag and 281 ‰ Cu weight percent [17,18]. Metallographic microscopy is one of the most powerful tools used to investigate alloys’ microstructures [17,19,20], but it requires local grinding and polishing prior to chemical etching and it is not effective for application to small coins that have been heavily exposed. Some powerful non-destructive methods are required in our case. XRD is a method that reveals the phase composition of alloy samples giving a proper qualitative characterization and allowing for a semi-quantitative analysis of the composition of binary systems [21,22]. It is often coupled with the SEM-EDS elemental investigation method due to the high precision of the elemental quantification related to the high-resolution imaging of the investigated site [22,23]. Modern SEM-EDS allows for the high-resolution complex elemental mapping of the microstructure, which in turn enables the visualization of the micro-structural constituents within the Ag–Cu binary system without polishing and chemical etching. This would be a great achievement for the present research. Therefore, we aim to use a combination of XRD and high-precision SEM-EDS analysis to elucidate the mysteries regarding the silver depreciation of the 3-Polkercoins produced during the reign of Sigismund III of Poland during 1619–1627.

## 2. Materials and Methods

### 2.1. Samples’ Description and Preparation

**Figure 1:**
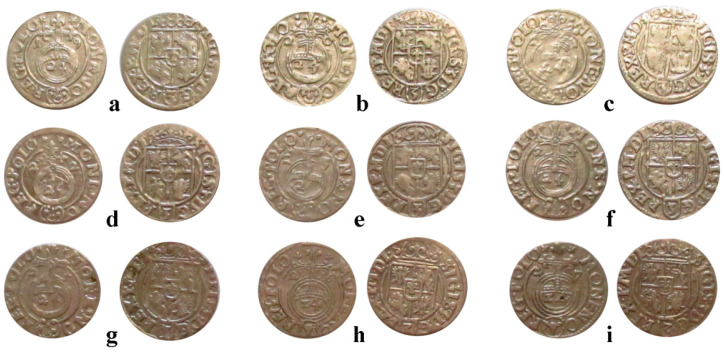
The3-Polker coins issued by Sigismund III of Poland investigated in the current research.Minting years: (**a**) 1619, (**b**) 1620, (**c**) 1621, (**d**) 1622, (**e**) 1623, (**f**) 1624, (**g**) 1625, (**h**) 1626, and (**i**) 1627. Coins’ dimensional characteristics are given in Table 1.

All samples investigated in the present research are 3-Polker coins issued by Sigismund III of Poland during 1619–1627 from the collection of Zalau Museum of History and Arts, Zalau City, Transylvania, Romania. Each coin is presented in Figure 1 with obverse and reverse sides grouped on the same indicative.

The coins were selected from several hoards and numismatic accumulations discovered in Transylvania (Salaj and Cluj counties) and hosted by the Zalau museum. The coins issued in 1619 and 1620 are from Salajeni Hoard; coins issued in 1621–1622 are from Verveghiu Hoard; coins issued in 1623 are from Hoard No. 1 of Zalau (the earliest 3-Polker hoard found in Transylvania); coins issued in 1624 and 1625 belong to the Mineu Hoard; the coins issued in 1626 and 1627 were selected from the Aghires Hoard (Cluj County). Each mentioned hoard contains only few issue years from the considered range. Therefore, we selected coins from five different hoards. It is noteworthy to mention that the earliest issue years for this currency (e.g., 1614–1618) are very rare in the hoards found in Transylvania and were not available for this study.

Coins’ physical characteristics such as mass, diameter, and thickness were measured and centralised in Table 1. Each coin was weighed on an electronic analytical balance with the precision of four digits; diameter and thickness were measured with a standard micrometer device.

Each coin was individually inspected in terms of their good preservation and absence of defects caused by long-term exposure to soil weathering; additionally, the coins were properly cleansed. Data in literature describe an alternative cleaning method using high-frequency cold plasma cleaning [24] to remove all impurities. However, the coins were previously restored and conserved according to the museum’s procedures. Current cleaning was effectuated using standard calcium bicarbonate detergent (15 min soaking and 5 min of gentle washing with cotton cloth)followed by intensive rinsing with bi-distilled water in order to cleansethe coin to properly expose the metallic surface to the non-destructive investigation methods.

### 2.2. Optical Microscopy

The general aspect of the coins’ surface was investigated by optical transmitted light microscopy using lateral light source dark-field observation on a Laboval 2 microscope, Carl Zeiss, Oberkochen, Germany, equipped with a digital capture camera Kodak 10 Mpx, Rochester, NY, USA.

Metallographic investigation was on an Olympus BX3M metallographic microscope, Shinjuku, Tokyo, Japan. The metallographic investigation was performed on 3-Polker coin fragments from years 1619 and 1625. Półtoraks’ broken fragments were discovered during archaeological digging. The year numerals“9” and“5” were observed on the right side of the cross, allowing for the identification of the issue years. The metallographic investigation was effectuated on the reverse side (the side with the numeral). The fragments were embedded into epoxy resin and successively grinded with abrasive discs with granulation of 800, 1200, 2000, and 4000 and followed by alumina polishing. They were chemically etched with Potassium Dichromate and Hydrochloric Acid for 45s and rinsed with bi-distilled water and soaked on the filter paper.

### 2.3. X-ray Diffraction (XRD)

X-ray diffraction (XRD) was effectuated on the coins’ reverse side (face with the years’ numerals) using a Bruker D8 Advance diffractometer with CuKα1 monochromatic radiation (λ = 1.54056 Å) at room temperature. The XRD patterns were recorded in the range 30–100 of 2-theta degree with a speed of 1 degree/minute. The phase identification was effectuated with Match software, Crystal Impact Company, Bonn, Germany, using the PDF database.

### 2.4. Scanning Electron Microscopy and Elemental Analysis (SEM-EDX)

SEM images were obtained on the coins’ reverse side (face with the year numerals), with a Hitachi SU8230 microscope, equipped with an Energy-Dispersive Spectroscopy (EDS) detector X-Max 1160 EDX (Oxford Instruments). The microscopic and elemental analyses wereconducted on at least three different spots on the coins’ surface.

## 3. Results

### 3.1. Optical Microscopy

**Figure 2:**
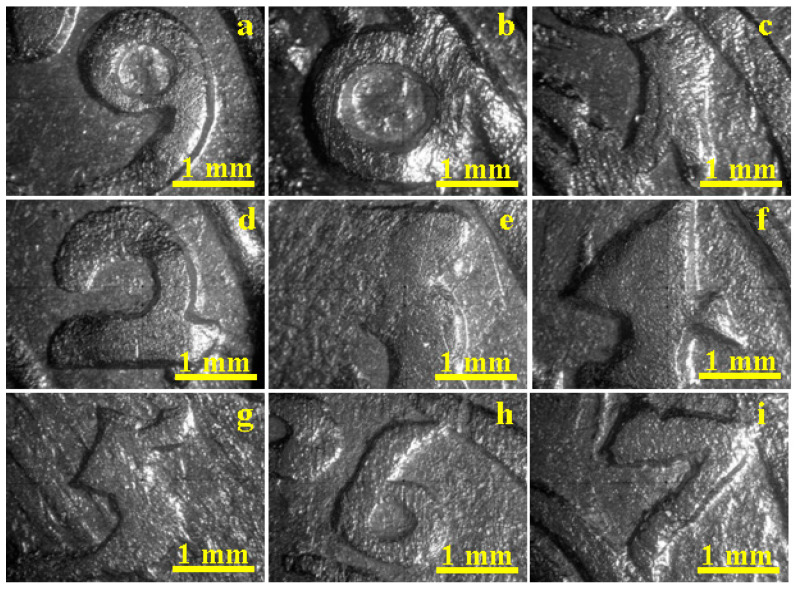
Dark field opticalmicroscopy images of the investigated 3-Polker coins from the minting years: (**a**) 1619, (**b**) 1620, (**c**) 1621, (**d**) 1622, (**e**) 1623, (**f**) 1624, (**g**) 1625, (**h**) 1626, and (**i**) 1627.

All the coins involved in present research were well-conserved in the museum collections preventing their surface depreciation. The cleaning procedure removed any traces of patina on their surface, there by allowing for the metal core to be observed. The success of the cleaning operation and the general aspect of the coins’ surfaces were monitored by dark field optical microscopy, as shown in Figure 2. Thus, the cleaning procedure removed all the impurities from all the coins from 1619 to 1627.

The lateral lighting used in the dark field observation enabled the better observation of the coins’ reliefs. There are two topographic planes on the coins: the upper one formed by the top of the inscriptions and the lower one formed by the base generated by the die surface. The wear from circulation affected the top of the inscription while the base irregularities are more related to the die quality.

The coin minted in 1619 presents a very good quality of the inscription without scratches and blunted details as well as a smooth base (Figure 2a). No traces of impurities such as dirt or oxide conglomerate are observed. It proves that the coin was engraved with very good quality dies and is less circulated, thereby preserving the engraved details.

The coins issued in 1620 and 1621 present some scratches on the top of their inscriptions, shown in Figure 2b,c, evidencing its more intense circulation and the good quality of the base, thus indicating that they were engraved with good quality dies. The coins issued in 1622 feature less wear from circulation but there are some irregularities on the base indicating an advanced usage of the dies (Figure 2d).

The numeral three is blunted on the left side of the coin from 1623 and presents significant scratches on the right, as shown in Figure 2e, due to intense wear. The base also presents blunted areas along with micro-structural irregularities. This indicates an intense usage of the dies when the coin was engraved combined with significant wear from circulation.

The coins issued between 1624 and 1627, show in Figure 2f–i, reveal a moderate degree of wear from circulation on the top of the inscription; the numerals observed are well-preserved and provide good details. Their base is affected by some pitches, which indicate an advanced usage of the dies when the coins were engraved. Some small darkened spots are observed at the inscription’s junction with the base for the coins from 1624, 1625, and 1627 (Figure 2f,g,i). These spots might be oxide traces; their nature will be revealed by XRD analysis.

**Figure 3:**
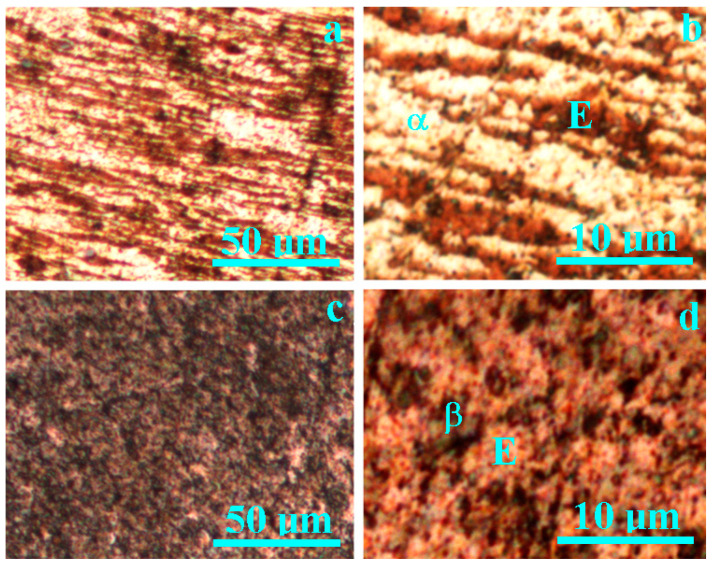
Metallographic microstructures of 3-Polker coin issued in 1619: (**a**) general view and (**b**) microstructure detail and of coin issued in 1625: (**c**) general view and (**d**) microstructure detail, where: E—eutectic, α—silver phase, and β—copper phase.

A metallographic investigation was conducted on some of the fragments of the three-Polker coins from 1619 and 1625 discovered in an archaeological dig. The obtained microstructures are presented in Figure 3. The coin issued in 1619 presents a typical microstructure of a good quality silver alloy, shown in Figure 3a,b. α phase grains representing pure silver and eutectic grains representing a mixture of about 71.9% Ag and 28.1% Cu were identified. The grains’ shape is elongated in the lamination direction of the silver plate used for coin blanks. This observed aspect is in good agreement with the data in the literature [25,26].

The coin issued in 1625 features a hypereutectic microstructure corresponding to a poor silver quality. Grains of a β phase (pure copper), mixed with eutectic Ag–Cu, were identified(Figure 3c,d). Fact is in good agreement with the data in literature [27,28]. The grain structure is very fragmented due to the intensive deformation during the plate lamination but does not show elongation. This is explained by the large presence of the β phase. It will be interesting to correlate these facts with the XRD investigation.

### 3.2. X-ray Diffraction (XRD)

**Figure 4:**
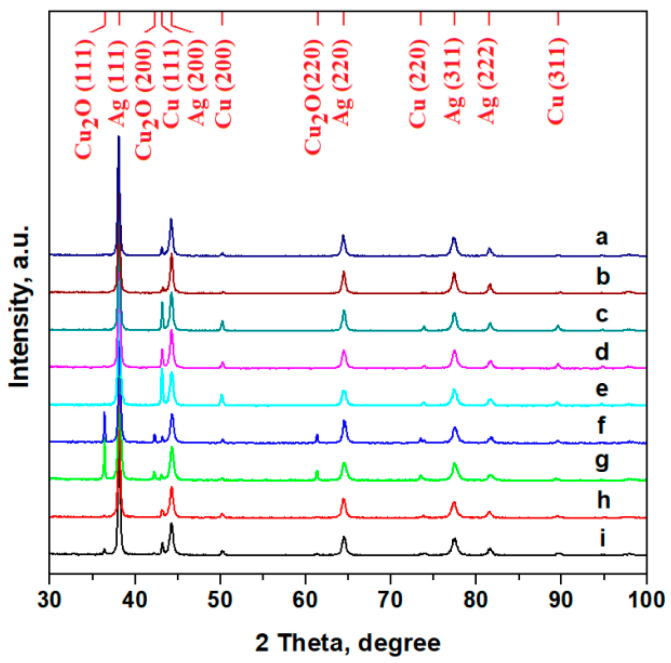
XRD spectra for the investigated coinsissued in: (a) 1619, (b) 1620, (c) 1621, (d) 1622, (e) 1623, (f) 1624, (g) 1625, (h)1626, and (i) 1627.

The XRD patterns of the investigated coins are presented in Figure 4. They feature very intense and narrow peaks corresponding to a crystalline state of the samples. The obtained XRD results prove that the coins correspond to the Ag–Cu binary system.

The samples were analysed by XRD, and it was found that they contain Ag (PDF:2871), Cu (PDF:89-2838), and some trace amounts of Cu_2_O (PDF:78-2076).The Reference Intensity Ratio (RIR) method was used to determine the percentage content of each phase. If we have two phases, *a* and *b,* then:

(1) $$\frac{I_{a}}{I_{b}}=\left(\frac{xa}{xb}\right)\left(\frac{cfa}{cfb}\right).$$

where *Ia* and *Ib* are the most intense diffraction peaks for phases a and b, respectively; *xa* and *xb* are the mass percentage concentrations for phases *a* and *b,* respectively; and *cfa*, *cfb* are the corundum factors for the two phases. The corundum factors are 18 for Ag, 8.86 for Cu, and 8.28 for Cu_2_O (cuprite). Using equation 1, the mass concentration ratio for the two phases can be determined. A phase analysis was performed using the RIR method and the results are presented in Table 2. The dominant peak corresponds to Ag in all cases but an increasein the Cu peaks was observed for certain issuing years such as the range from 1621–1625.

Several Cu_2_O peaks were identified for the coins issued in 1624, 1625, and 1627, which are correlated with the very small dark dots observed by optical microscopy in Figure 1. The presence of cuprite affects the copper content determined by the RIR method from the XRD patterns but does not affect the silver content. The results sustain a significant depreciation of the silver title between 1621 and 1625, a fact that is in good agreement with the historical information about the monetary inflation but that does not agree with the very low title mentioned in the historical sources.

The data in the literature mention the cuprite formation on the restored coins after a long-time storage [29]. Therefore, a fresh cleaning is required every time a coin is subjected to a material analysis. However, a small amount of copper oxides such as cuprite and tenorite may occur in the XRD patterns, for example, as in one of our previous studies on a freshly cleaned lot of Dyrrachium drachmas from the Cehei hoard [30].

### 3.3. Scanning Electron Microscopy and Elemental Analysis (SEM-EDX)

The SEM investigation associated with the elemental analysis was very complex and investigated several morphologic features of the coins’ surfaces. We found some very rich silver areas on the bases of the coins’ surfaces that might have occurred due to the initial restoration effectuated at the moment of the hoard’s preservation in the museum treasury or due to some actions from sometime in the past. This aspect will be expanded upon and discussed in a further paper. The tops of the inscriptions are considerably worn compared to the bases and further expose the material inside the coin, which is ideal for the morphological observation as well as for the EDS spectroscopy.

**Figure 5:**
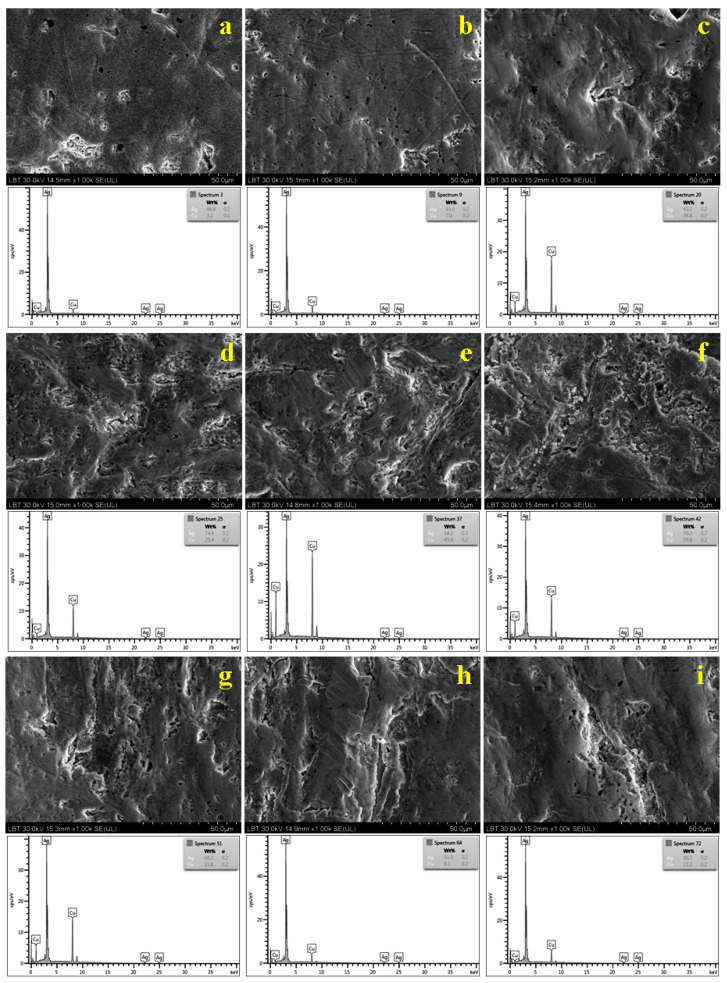
SEM images and EDS spectra resultingfrom the investigated 3-Polker coins issued in: (**a**) 1619, (**b**) 1620, (**c**) 1621, (**d**) 1622, (**e**) 1623, (**f**) 1624, (**g**) 1625, (**h**) 1626, and (**i**) 1627.

Figure 5a reveals the morphology of the 1619 coin with a uniform surface and a compact microstructure. Small pores were observed with diameters in the range of 1.5 to 5 µm. These are related tothe poor-quality workmanship of the hammered coins’ manufactured in medieval times, which is often associated with micro-mineral contaminants on the dies’ surfaces. Several corrugations with dendrite shapes and lamellar inner structures were observed. These are related to the lamellar Ag–Cu eutectic grains, which are more predisposed to the mechanical erosion.. A similar microstructure is observed for the coin issued in 1620, shown in Figure 5b, which displays smaller pores with diametersbetween 1–2.5 µm and some superficial scratches related to significantly intense circulation.

The morphological aspect is significantly changed starting from 1621 (Figure 5c). The surface is not very uniform, which indicates that it was more affected by soil weathering. There appear to be a few pores with diameters between 2 to 8 µm and enlarged corrugated dendrite areas related to the eutectic presence. The microstructural change is more likely caused by the increase in the amount of copper.

The microstructures of the coins issued from 1621 to 1625 present similar aspects as those observed in Figure 5d–e. There are relatively few pores with an equivalent diameter in the range of 1 to 3 µm. The surfaces are relatively irregular due to the alternation of the compact and lamellar grains corresponding to a typical hypereutectic microstructure [27].

Another microstructural change was observed for the coins issued in 1626 and 1627, shown in Figure 5h,i. Their morphologiesclosely resemble those observed for the coins issued in 1619 and 1620. Large compact grains with wear marks and eutectic dendrite— partially corrugated due to the coins’ circulation—were observed. This might be a microstructural clue regarding the silver title’s restoration.

The microstructural observations are coupled with the XDS spectra obtained for the observed surface. The resulting elemental composition is summarised in Table 3. It is in good agreement with the XRD results in Table 2.

**Figure 6:**
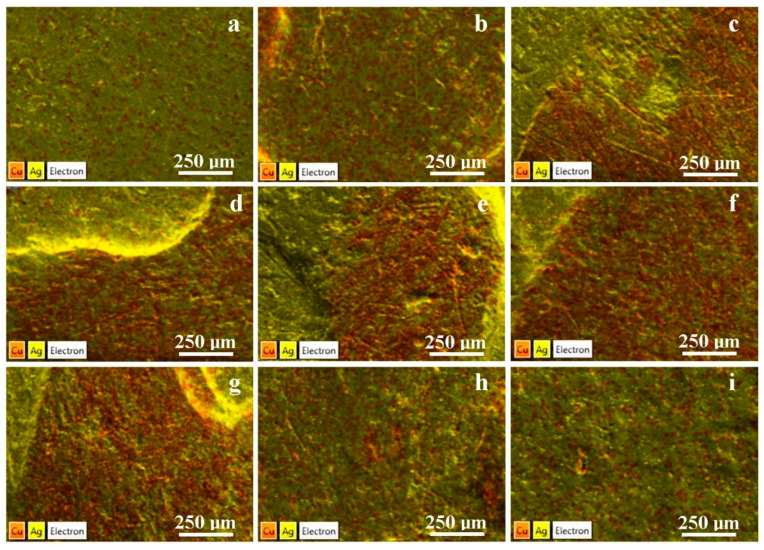
Elemental maps of the 3-Polker coins issued in: (**a**) 1619, (**b**) 1620, (**c**) 1621, (**d**) 1622, (**e**) 1623, (**f**) 1624, (**g**) 1625, (**h**) 1626, and (**i**) 1627.

The coins issued in 1619 and 1620 have a good silver title, even better than the reference for the German Thaler, which is 83.5%. These are situated in the hypoeutectic domain of the Ag–Cu binary system from the metallographic point of view. This means that the microstructure containsα phase and eutectic grains. This fact is sustained by the elemental map in Figure 6a,b: the α-phase grains present a green aspect dotted with yellow spots due to the rich silver amount and the eutectic grains present brown spots due to the presence of copper.This result corresponds to the metallographic observation in Figure 3b.

A significant depreciation of the silver title was found for the coins issued from 1621 to 1625. The coin issued in 1622 is very close to a eutectic composition, which means that the microstructure contains a large number of eutectic grains and only a few grains of the α phase. Thisfact is sustained by the EDS map in Figure 6c, wherein the copper amount corresponding to the eutectic region is intense and tends to form compact dendrite areas.

The othercoins issued in 1621, 1623, 1624, and 1625 are situated in the hypereutectic domain of the Ag–Cu binary system. A significant amount of β copper-rich phase grains mixed with eutectic grains occurs in these coins. This fact is sustained by the EDS maps presented in Figure 6d–g. We observed compact areas (brown-coloured) with a dendrite shape, which correspond to the β phase grains. The green areas dotted with small brown spots correspond to the eutectic grains. The elemental map of the coin from 1625, shown in Figure 6b, corresponds to the metallographic aspect evidenced in Figure 3d.

Some yellow bands can be observed in the EDS maps in Figure 6d,e,g. These bands are not situated on the top of the inscription but rather appear in some areas situated at the base of the inscription and are silver-rich areas which correspond to the silver’s segregation. These aspects are not related to the bulk material of the coins. Rather, they are related to a possible physicochemical reaction between the preservation compounds and the coin surface. The compound was certainly removed by the cleaning procedures, but it seems that some silver segregation still remains in the mentioned places. These facts require more detailed investigation, which will be the subject of a further article.

The coins issued in 1626 and 1627 present a high amount of silver situated in the hypoeutectic region of the Ag–Cu binary system and present in the elemental maps—shown in Figure 6h,i—with a strong resemblance to the observation made for the coins issued in 1619 and 1620. These facts indicate that the silver title was properly restored in 1626 when the mint master was changed.

The depreciated coins may have traces of elements such as Sn, Pb, and Zn, as impurities resulted from the improper metallurgical process or were deliberately placed in the composition to falsify the silver content. The elemental analysis was effectuated with high accuracy, and trace elements such as Sn, Pb, and Zn were searched for but not detected. This result is in good agreement with the XRD observation, where no odd phases were detected.

**Figure 7:**
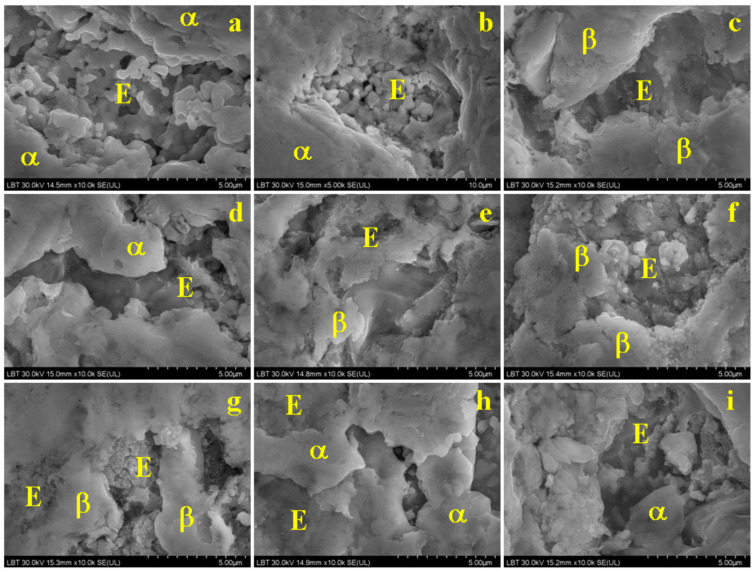
SEMmorphologic details revealed on the surface of investigated 3-Polker coins issued in: (**a**) 1619, (**b**) 1620, (**c**) 1621, (**d**) 1622, (**e**) 1623, (**f**) 1624, (**g**) 1625, (**h**) 1626, and (**i**) 1627, where E—eutectic, α—silver phase, and β—copper phase.

The EDS spectra along with the distribution maps allow forthe identification ofthe microstructures’ constituent grains without polishing and chemical etching but by obtainingthe proper details from the secondary electron images. The eutectic grains within the good silver coins have an elongated dendritic shape with a fine lamellar structure, but the lamellas are broken due to the coins’ surface wear (Figure 7a,b,h,i). They are surrounded by very compact α-phase grains that also have an elongated shape.

The eutectic grains are more affected by the wear in the depreciated coins (Figure 7c,e–g). The lamellas are also affected, implying a significant material loss corresponding to the formation of local depressions. These are surrounded by compact β-phase grains, which also present an elongated shape and wear marks on their surface.

A special microstructure was revealed for the coin issued in 1622 because it is situated in the hypoeutectic domain but very close to a eutectic composition. Therefore, the eutectic grains are very numerous, their shape is dendritic and elongated, and many of them are affected by the coins’ surface wear, as shown in Figure 7d. Some compact α-grains with diameters below 5 µm can be observed. They are primarily crystallized from the melted alloy during cooling, while the eutectic grainsare formed when the temperature decreases to about 779 °C.

**Figure 8:**
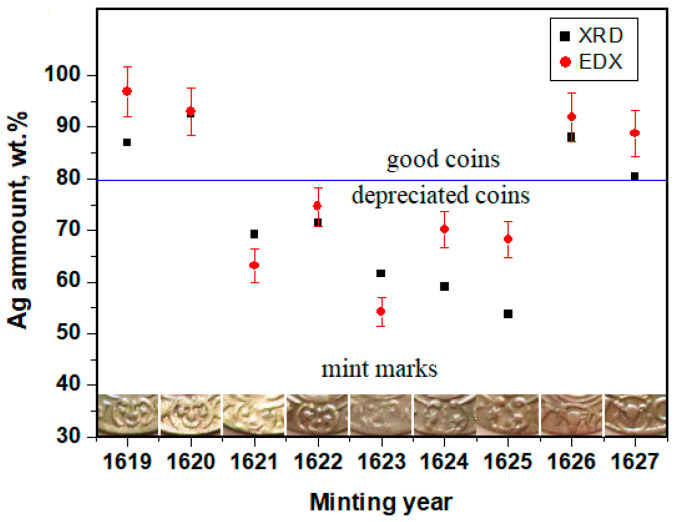
Silver content variation during the minting time evidenced by XRD and SEM-EDS compared with the aspect of the mint marks.

The coins’ silver amount values measured by XRD and SEM–EDX are displayed in Figure 8. The border line between good and depreciated coins is on the title of 80% Ag. It is clear from the results that the coins issued in 1619, 1620, 1626, and 1627 are made of good silver.

The coins issued in 1621, 1622, 1623, 1624, and 1625 have depreciated silver titles. Despite the significant silver portion decreasing the observed depreciation, this effect is not as significant as expected based on the historical information. The mint mark of each coin was also placed in Figure 8 because some of the graphical aspects tend to be correlated with the depreciation. The connection between the reduced silver title and mintmark variation will be discussed below.

## 4. Discussion

The coins investigated in the present research are very interesting because they have corresponding currencies in two monetary systems based on the silver title. Theirface value and nominal equivalence are given by expression (2):

(2) $$1Półtorak\left(e.g.3\;Polker\right)=3\;denars=1.5\;Gross=1.5\;Kreutzer=\frac{1}{24}Thaler\;$$

where *Denars* are a Hungarian small silver denomination, *Gross* are a typical Polish small silver denomination, and *Kreutzer* are the imperial small silver denomination (of the Holy Roman Empire). The silver standard at that time was the *Thaler* of 28.83 g, containing 24.31 g of fine silver according Gumovski [5], which is in good agreement with Toma [4]. It corresponds to a title of 84.3%; consequentially, a 3-Polker coin must have a weight of 1.20 g of silver alloy with a title of84.3% corresponding to 1.01 g of fine silver as a standard reference.

The correlation of the data in Table 1, Table 2 and Table 3 demonstrate that coins issued in 1619 and 1620 have a title significantly higher than the standard reference and a weight in excess of about 0.1g and 0.16g. In consequence, these coins fit the standard mentioned by Gumovski [5]. The coins issued in 1626 and 1627 have a weight and title around the standard value, proving their quality. The coins issued from 1621 to 1625 are lighter with about 0.12 to 0.2g and their silver title is situated in the range of 63.2–74.6%, a fact which indicates a certain depreciation compared to the standard.

The mintmark of Nicolas Danilowicz, Grand Treasurer of the Crown, features a horizontal moon crescent with a vertical arrow that separates two stars and is placed in round brackets below the cross orb on the coin’s obverse side. The stars are horizontally aligned for the good title coins and the right star is placed slightly lower for all depreciated coins. As a supposition, this might be a sign for anexpert to easily identify the depreciated coins.

Therefore, the coin collector’s supposition of the continuous depreciation of the silver title with the issuing year has been rejected by the obtained results. The material evidence indicates that the depreciation is situated in the period of 1621–1625, which is in good agreement with the historical data but also disagrees with the mentioned title of 40.6% [4,5]. Our findings confirm the theory of historian Buza János [8] that the 3-Polker coins issued by Sigismund III of Poland cannot be depreciated coins with a title of 40.6%.

This historical contradiction resides in the medieval expression that “copper-rich Polturak” came from Silesia, which describes coins with a silver title about 40.6%. Silesia was certainly not a Polish possession and the 3-Polker coins afflux from this region is unlikely. The explanation resides in the medieval crisis known as,“Kipper- und Wipperzeit”, which starts in 1619 along with the 30-years war [31,32]. The small coin title was debased from the nominal value of 84.3% to about 40.6% in several states such as Silesia. However, they did not debase their own coins, preferring to forge other countries’ coins, such as the Polish 3-Polker, and passing them on to other territories. Massive inflation arose in those conditions and the face value of the coins was not respected. Each individual coin was inspected and weighed; the good ones were kept for the treasury and the depreciated ones were discarded.

Many of these coins arrived in Transylvania and were subjected to the Cluj and Bistrita diet regulations in 1622. In addition, the Transylvanian issues of Gabriel Bethlen were debased during 1619–1623, causing monetary confusion [4]. In such conditions, people managed to identify good 3-Polker coins issued by Poland and treasure them and thus try to circumvent the use of bad coins. It seems that the Kipper- und Wipperzeit crisis had less of an effect on the original Polish coinsissued during 1621–1625, being slowly and weakly depreciated compared to the Transylvanian and Silesian coins, which were strongly depreciated. This fact caused the preference for the hoarding of 3-Polker coins along with other denominations such as the triple and sextuple gross issued in Poland at that time.

The forged 3-Polker coins with a title of 40.6% were never found in a hoard and they are very rare in Transylvania because they were collected and melted down during the monetary reform effectuated by Gabriel Bethlen in 1626. Our findings prove that the silver title restoration for the Polish 3-Polker starts in 1626 and continues until 1627 when the issue stops because the triple and sextuple gross become more popular.

## 5. Conclusions

The materials science investigation (XRD and SEM –EDX) effectuated on the 3-Polker coins issued by Sigismund III Vasa, King of Poland during 1619 and 1627 evidenced a certain depreciation of the silver title from about 84.3% to a range of 63.2–74.6% with respect to the coins issued between 1621–1625. Therefore, the material information confirms the historical data about the inflation during 1621–1625 but the depreciated title is not as low as that recorded by historical sources. This fact is sustained by the archaeological records that prove that the investigated coins were hoarded, which means that they were considered good at that time. An important conclusion derived from the observation within the current research is that the “copper rich Półtorak” is definitely not equivalent to the 3-Polker coins issued by Sigismund III of Poland. The coins issued in 1619, 1620, 1626, and 1627 have a very good title, with most of them exceeding 83.5%, proving the title’s restoration after 1626.

## Figures and Tables

**Table 1:** Coins physical characteristics.

Minting Year	Grand Treasurer of the Crown	Mass, g	Diameter, mm	Tickness, mm
1619	Nicolas Danilowicz	1.3679	19.84	0.65
1620	Nicolas Danilowicz	1.3054	19.57	0.65
1621	Nicolas Danilowicz	1.0341	19.38	0.50
1622	Nicolas Danilowicz	1.0279	19.32	0.54
1623	Nicolas Danilowicz	1.0842	19.28	0.53
1624	Nicolas Danilowicz	0.9773	19.67	0.55
1625	Nicolas Danilowicz	1.0618	19.23	0.59
1626	Hermann Ligenza	1.2629	19.17	0.59
1627	Hermann Ligenza	1.1632	19.33	0.58

Note: All coins were minted at Bydgoszcz Mint.

**Table 2:** Phase composition of the investigated 3-Polker coins.

Minting Year	Ag (%)	Cu (%)	Cu_2_O (%)
1619	86.97	13.03	-
1620	92.49	7.51	-
1621	69.15	30.65	-
1622	71.36	28.64	-
1623	61.61	38.39	-
1624	59.14	6.86	34.06
1625	53.76	5.00	41.24
1626	88.03	11.97	-
1627	80.40	15.35	4.25

**Table 3:** Elemental compositions of the investigated 3-Polker coins.

Minting Year	Ag		Cu		Traces
	Wt.%	S.D.	Wt.%	S.D.	Wt.%
1619	96.8	4.84	3.2	0.22	undetectable
1620	93.0	4.65	7.0	0.49	undetectable
1621	63.2	3.16	36.8	2.57	undetectable
1622	74.6	3.73	25.4	1.77	undetectable
1623	54.2	2.71	45.8	3.20	undetectable
1624	70.2	3.51	29.8	2.08	undetectable
1625	68.2	3.41	31.8	2.22	undetectable
1626	91.9	4.59	8.1	0.56	undetectable
1627	88.7	4.43	11.3	0.79	undetectable

## Data Availability

Data sharing is not applicable.
